# The role of extracellular vesicles in the transport and regulation of novel inflammatory mediators in IBD and its associated CRC

**DOI:** 10.3389/fcell.2026.1760517

**Published:** 2026-03-31

**Authors:** Chuxin Zhang, Xiaohua Tang, Sixuan Chen, Francis Atim Akanyibah, Fei Mao

**Affiliations:** 1 Department of Laboratory Medicine, School of Medicine, Jiangsu University, Zhenjiang, Jiangsu, China; 2 Department of Laboratory Medicine, The Affiliated People’s Hospital, Jiangsu University, Zhenjiang, Jiangsu, China; 3 The People’s Hospital of Danyang, Affiliated Danyang Hospital of Nantong University, Zhenjiang, Jiangsu, China; 4 Department of Preventive Medicine and Public Health Laboratory Science, School of Medicine, Jiangsu University, Zhenjiang, Jiangsu, China

**Keywords:** colorectal cancer, extracellular vesicle, inflammatory bowel disease, mesenchymal stem cell-derived EVs, novel inflammatory mediators, therapy

## Abstract

Inflammatory bowel disease (IBD), comprising ulcerative colitis (UC) and Crohn’s disease (CD), is a chronic inflammation of the gut characterized by an imbalance in the intestinal microbiome and ecology. IBD raises the risk of developing colorectal cancer (CRC). CRC is one of the most commonly diagnosed cancers in the world, with high incidence rates. Extracellular vesicles (EVs) play a crucial role in intercellular communication and are vital for maintaining intestinal homeostasis. Recent research highlights novel inflammatory mediators, such as specialized pro-resolving mediators (SPMs), damage-associated molecular patterns (DAMPs), alarmins, non-coding RNAs (ncRNAs), and metabolic intermediates, as crucial in the pathophysiology of IBD and CRC. These novel inflammatory mediators are transported by EVs, influencing the pathogenesis of IBD and associated CRC. Therefore, this article examines the role of novel inflammatory mediators transported by EVs in the pathogenesis of IBD and related CRC, as well as the interaction between EVs and the tumor microenvironment. We also review new research on EV use as a diagnostic indicator and on the potential of EVs, such as mesenchymal stem cell-derived EVs (MSC-EVs), as therapeutic delivery channels for cancer treatment targeting unique inflammatory mediators.

## 1 Introduction

Inflammatory bowel disease (IBD) is a group of persistent, nonspecific inflammatory gut disorders caused by a combination of immunological, genetic, environmental, and other factors. IBD has two subtypes, including ulcerative colitis (UC) and Crohn’s disease (CD), which are characterized by debilitating and chronic recurring and resolving inflammation in the gut and gastrointestinal system, respectively (Ocansey et al., 2020). The incidence of IBD has increased dramatically in both industrialized and developing countries, and people are now being diagnosed at an increasing age (Agrawal and Jess, 2022). Compared with low-income regions, high-income countries have seen a decline in IBD-CRC incidence due to improved surveillance systems and advanced disease treatment strategies. However, underreporting in low- and middle-income areas masked the global burden (Kapadia et al., 2025). Although the cause and pathophysiology of IBD are complex and unknown, recent research has focused on the interplay between chronic intestinal inflammation and the intestinal milieu. In IBD patients, the increased production of extracellular vesicles (EVs) and changes in their contents play a dual role in the intestinal environment, serving both pro-inflammatory and anti-inflammatory functions by influencing macrophage polarization and aiding microbial rebuilding (Shen et al., 2022).

IBD is a persistent, recurring condition, and the cumulative effects of chronic inflammation on long-term IBD patients are connected with an increased risk of CRC, referred to as colitis-associated colorectal cancer (CAC) (Xia et al., 2023). Compared to sporadic CRC, IBD-driven tumors frequently manifest with a distinct mutation pattern, extremely heterogeneous hyperplasia, and worse survival rates (Choi et al., 2017). The major mechanism by which chronic inflammation drives IBD-associated CRC involves triggering inflammation and accelerating the repetitive cycles of epithelial injury and repair, which promotes mutagenesis (Porter et al., 2021). Therefore, understanding the mechanism of association between IBD and CRC is vital for prevention and treatment.

**FIGURE 1:**
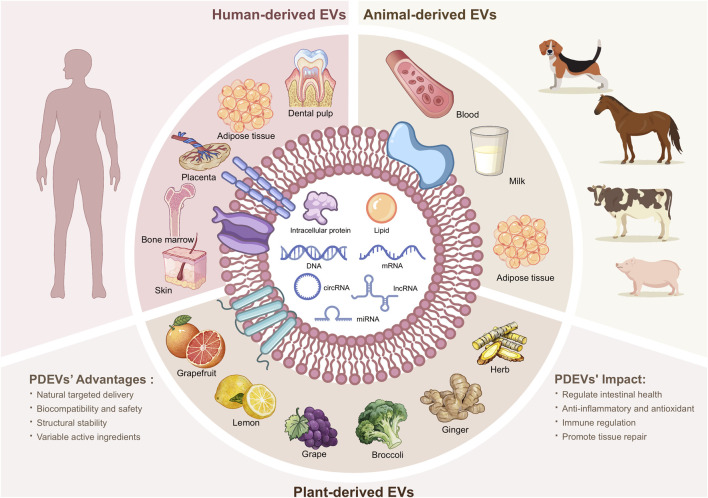
Different origins of widely studied EVs. Presently, in-depth research on EV primarily focuses on the human body, animals, and plants. EVs can carry a variety of substances, including intracellular proteins, lipids, DNA, RNA, membrane proteins, and other signaling molecules. PDEV, plant-derived extracellular vesicle; DNA, deoxyribonucleic acid; RNA, ribonucleic acid; circRNA, circular RNA; lncRNA, long non-coding RNA; miRNA, microRNA.

EVs are structures composed of proteins and phospholipids that cells continuously release. They can be found in both small particles measuring 30 to 200 nm and larger, micron-sized particles (Shen et al., 2022). The sources of EV are vast and varied, and almost all cell types can produce them (Figure 1). The well-studied origins of EVs are shown in Figure 1. As crucial participants in intercellular communication, they transport not only membrane proteins and lipids but also ribonucleic acid (RNA), cytoplasmic proteins, and other signaling chemicals to the recipient cell. Exosomes, microvesicles, and apoptotic bodies are the three primary groups of EVs based on their biogenesis (Shen et al., 2022). They are crucial for normal physiological functions such as blood coagulation, immunological surveillance, tissue repair, and stem cell maintenance, as well as for the pathology of various illnesses, including cancer and inflammation (S et al., 2013). Numerous studies have reported that dysbiosis of the gut microbiota in patients with IBD, along with the aberrant metabolism of intestinal epithelial cells (IECs), is associated with alterations in the quantity of EV cargo (Chang et al., 2020). Moreover, in individuals with gastrointestinal malignancy, exosome-loaded microRNAs (miRNAs) and proteins have markedly different expression patterns (Matsumura et al., 2015).

The pathogenesis of IBD and CRC has long focused on the role of classical cytokines and chemokines. However, novel inflammatory mediators have gone beyond these classical molecules and become emerging core participants in the pathogenesis of IBD and CRC. They regulate inflammation, tissue damage, and tumor development either positively or negatively through various mechanisms, including SPMs, alarmins, DAMPs, non-coding RNAs, and metabolic intermediates (Gobbetti et al., 2017; Santana et al., 2024). Therefore, research on novel inflammatory mediators is expected to overcome the limitations of traditional therapies and restore immune stability. Hence, this review examines the role of EVs from various origins in IBD and their progression to CRC, focusing on the transport and regulation of specific inflammatory molecules. It also provides a research direction and potential therapeutic target for IBD treatment in the future by further investigating the interaction of novel inflammatory mediators and EVs on the gut, exploring the therapeutic potential of targeting EVs and novel inflammatory mediators in IBD and CRC, and providing new strategies for precision intervention of IBD and IBD-associated CRC. Also, all forms of EVs, including exosomes, tiny extracellular vesicles, and small vesicles, will be included in the review, due to the paucity of research on novel inflammatory mediators in EVs.

## 2 Extracellular vesicles as key players in IBD pathogenesis

### 2.1 EVs in the inflamed intestinal microenvironment

**FIGURE 2:**
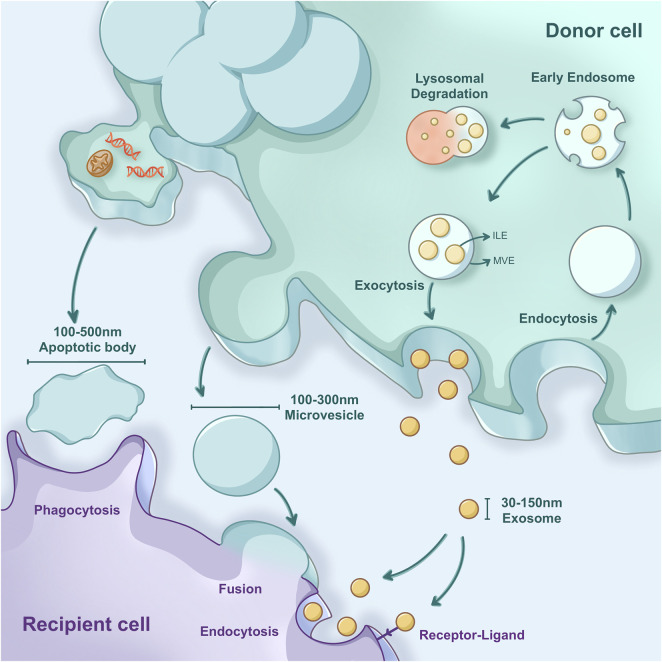
Classification of EVs is based on their biogenesis, release, and uptake. EVs comprise apoptotic bodies, microvesicles, and exosomes. Exosomes are produced by budding from multivesicular bodies. Microvesicles are generated intracellularly from the extracellular membrane. Apoptotic bodies form when cells fragment during cell death. EV uptake mechanisms include direct fusion of EV membranes with the plasma membrane, resulting in the discharge of contents into recipient cells, as well as endocytosis, phagocytosis, and macropinocytosis.

EVs in IBD mainly come from immunological cells (dendritic cells (DCs), macrophages, T cells, and B cells), IECs, endothelial cells, and fibroblasts (Shen et al., 2022). EVs are classified into three types based on their biogenesis: exosomes, tiny vesicles, and apoptotic bodies (Figure 2). EVs in the gastrointestinal system contain various molecular components depending on their progenitor cells, which determine EVs’ activity (Li et al., 2023). Therefore, measuring signature molecules can identify the source of EVs. In the physiological environment, IECs secrete EVs from the apical or basolateral surfaces, and these EVs contain immunomodulatory molecules expressed on their surface, such as major histocompatibility complex (MHC)-I, MHC-II, and human leukocyte antigen-DM (HLA-DM) (Mallegol et al., 2007). They mainly interact with DCs to improve antigen presentation and maintain intestinal homeostasis; under inflammatory conditions, their expression levels are significantly higher than those of basic molecules (Mallegol et al., 2007). EVs produced by IECs expressing integrin αβ6 can activate regulatory T (Treg) cells and promote immune tolerance (Chen et al., 2011). However, in the inflammatory environment, the intestinal microenvironment promotes cellular stress and tissue inflammation by altering the biogenesis and content of EVs (Tkach et al., 2017). EVs are a great way to transport particular chemicals to recipient cells. Hence, inflammatory mediators, the major players in IBD, are contained by EVs and gradually delivered to target cells in the gut mucosa, where they execute specialized functions. These functions include modulating immunological responses, maintaining the gut barrier integrity, and influencing the intestinal flora in IBD.

### 2.2 Regulation of inflammatory responses by EVs in IBD

In general, the anti-inflammatory or pro-inflammatory functions of EVs are determined by their different cellular origins and cargos (Li et al., 2023). For individual EV, there is no switch between anti-inflammatory and pro-inflammatory effects. In fact, a substantial number of EVs from various origins interact with target cells and the intestinal microenvironment, forming a complex network that ultimately manifests as pro-inflammatory or anti-inflammatory phenotypes (Shen et al., 2022). In conditions of barrier impairment or dysbiosis (Camilleri et al., 2012; Imai et al., 2019), the gut tends to have a pro-inflammatory phenotype. When the intestinal barrier is intact and the microbiome is healthy (Leoni et al., 2015; Jacobs et al., 2016), the gut tends to exhibit an anti-inflammatory phenotype.

#### 2.2.1 EVs as pro-inflammatory signals

EVs regulate immune responses by transporting inflammatory mediators and other substances between immune cells. The proliferation of MHC I, MHC II, and HLA-DM, which occurs on the surface of EVs in an inflammatory state, can act as pro-inflammatory signals to activate a wider range of CD8^+^ and CD4^+^ T cells and release IFN-γ, leading to a more intense inflammatory response (Mallegol et al., 2007). In addition to surface markers, pro-inflammatory signals can also be released from within EVs. A study found that EVs released from the intestinal inflammation site in IBD patients exhibit a unique protein and mRNA profile compared with those from normal individuals (Ocansey et al., 2020). EVs from IBD patients with a high endoscopic score (≥1) contained significantly higher mRNA and protein levels of interleukin (IL)-6, IL-8, IL-17, and tumor necrosis factor (TNF)-α than EVs from healthy controls. Additionally, these EVs also showed pro-inflammatory effects on colon epithelial cells *in vitro* (Chen L. et al., 2023). Among the inflammatory cytokines, TNF-α promotes apoptosis and inflammation in IECs (Zhang C. et al., 2025); however, IFN-γ activates macrophages and neutrophils and boosts immune-cell recruitment by activating adhesion molecules on intraepithelial cells (Li et al., 2019). IL-17 promotes the release of IL-8 from epithelial cells, stimulating the recruitment of neutrophils and Th17 cells to inflamed tissues (Jiang et al., 2023). Nevertheless, the lack of IL-17 exacerbates DSS-induced colitis, suggesting that IL-17 also has a beneficial effect (Strober et al., 2002). In general, EVs act as pro-inflammatory signals by activating immunological cells through the transfer of inflammatory mediators, promoting cytokine production, and ultimately recruiting immune cells.

#### 2.2.2 EVs as anti-inflammatory signals

As anti-inflammatory signals, EVs are usually derived from anti-inflammatory cells or contain regulatory molecules. For example, M2b macrophages release a variety of anti-inflammatory mediators, such as transforming growth factor (TGF)-β, IL-10, C-C motif ligand 1 (CCL1), CCL17, and others. These anti-inflammatory mediators inhibit the activation of immune cells, thereby promoting tissue repair and regeneration (Hu et al., 2021). As a result, using EVs to deliver modulating molecules to treat intestinal inflammation has emerged as a viable method. Therapy with exosomes containing IL-10 led to a significant elevation of IL-10 mRNA levels in intestinal tissues and in Tregs in the lamina propria, suppressing acute 2,4,6-trinitrobenzene sulfonic acid (TNBS)-induced colitis (Yang et al., 2010). Under physiological conditions, IECs secrete TGF-β1-dependent EVs with immunosuppressive activity. These EVs can reduce the severity of dextran sulfate sodium (DSS)-induced IBD in mice by stimulating Tregs and immunosuppressive DCs (Jiang et al., 2016). Mesenchymal stem cell-derived extracellular vesicles (MSC-EVs) can also effectively alleviate gut inflammation by inhibiting mediators that promote inflammation (e.g., TNF-α, IL-6, IL-1β) and enhancing anti-inflammatory agents (e.g., IL-10, IL-4) (Clua-Ferré et al., 2024). However, extensive experimental and clinical research is required before MSC-EVs may be used as a successful treatment regimen for IBD and related CRC.

#### 2.2.3 The context-dependent and cell-type-specific effects of EVs in IBD

It is worth mentioning that the function of EVs in IBD depends on specific environmental conditions and cell types. The predominant focus of current immunological research is on DCs, T cells, and macrophages.

T cells, particularly Th17 cells (pro-inflammatory) and Tregs (anti-inflammatory), play an important role in adaptive immunological reactions and are required for IBD pathogenesis. A loss of balance between Treg and Th17 cells is the primary cause of gut tolerance dysfunction, leading to the establishment and progression of IBD (Zhang S. et al., 2024). Restoring the normal Th17/Treg ratio helps restore the intestinal microenvironment. In a mouse colitis model, exosomes derived from olfactory ecto-MSCs reduced the immune response by suppressing Th17 and Th1 cell growth while increasing Treg cell production (Tian et al., 2020). Studies have found that mice with blocked IL-17 exhibited more severe disease and symptoms of DSS-induced colitis. Interestingly, mice lacking interleukin-17 receptor (IL-17R) or treated with an interleukin-17 receptor immunoglobulin (IL-17R-Ig) fusion protein showed reduced IBD progression following TNBS induction. The dual role of IL-17 in maintaining the intestinal barrier has been indicated earlier, suggesting that its expression may depend on specific environmental and local inflammatory states within the microenvironment (Jiang et al., 2023; Strober et al., 2002).

Macrophages are closely associated with EV and IBD, and macrophage infiltration is often observed in the inflamed sites of IBD patients, altering the numbers and proportions of CD4^+^ T cell populations. Macrophages differentiate into two types based on their environment: pro-inflammatory M1 macrophages and anti-inflammatory M2 macrophages (Luo et al., 2024). In the pathogenesis of IBD, the imbalance between M1/M2 macrophages, driven by dysregulation of macrophage plasticity, is a critical factor. As key mediators of intercellular communication, EVs regulate macrophage polarization by delivering specific cargo. Intestinal macrophages in IBD patients exhibit a higher proportion of M1-like phenotypes, with surface markers of clusters of differentiation (CD)11c, CC chemokine receptor (CCR)2, HLA-DR, CD64, CD206, and CD209. They can produce inflammatory substances such as IL-23 and TNF-α, increase Th17, and contribute to microbial dysbiosis (Chang et al., 2020). Wei et al. demonstrated that adipocytes secrete pro-inflammatory EV-miR-155 into the blood circulation, which is then absorbed by macrophages in the intestine. These macrophages undergo M1-like polarization, thereby inducing chronic intestinal inflammation (Wei et al., 2020). Moreover, numerous studies indicate that promoting macrophage polarization toward an M2 phenotype is a promising therapeutic strategy for IBD. Compared with traditional IL-4, IL-10, and miRNAs that induce M2 polarization, EVs can be specifically recognized by receptor cells, thereby enabling more precise induction of macrophage M2 polarization. MSC-EV has been widely used, with successful transplantation in IBD animal models, providing a theoretical foundation for future clinical applications. By polarizing M2b macrophages in IBD colitis models, systemically delivered human bone marrow MSC-derived exosomes significantly reduce inflammation without inducing intestinal fibrosis. A study demonstrated that adipose tissue-derived mesenchymal stem/stromal cell (ASC)-derived EVs modulate the intestinal immune environment by reversing macrophage polarization, thereby effectively alleviating the severity of colitis. Tumor necrosis factor-α-stimulated gene/protein-6 (TSG-6) plays a key role in the polarization of macrophages from M1-like to M2-like phenotypes in the colon (An et al., 2020). Polarized M2b macrophage exosomes boosted Treg cells and suppressed pro-inflammatory cytokines, including IL-1β, IL-17A, and IL-6 (Yang et al., 2019).

DCs are antigen-presenting cells (APCs) that contain MHC-I and MHC-II molecules on their surfaces, which activate T cells (naive) and promote IBD progression (Darrasse-Jèze et al., 2009). DC-derived EVs influence IBD progression via immune regulation. Therefore, inhibition of DC activation can induce immune tolerance and regulate Treg activation. It has been documented that EVs tend to localize in the intestinal tract, specifically in association with epithelial cell adhesion molecule (EpCAM) (Chen G. et al., 2021). Inhibition of EpCAM expression in the colon exacerbates murine IBD, and the protective effect of EVs from IECs with decreased EpCAM expression on murine IBD is impaired (Jiang et al., 2016). In addition, efficient immune responses to EVs from migratory DCs require local secretion in lymphoid tissues (Lindenbergh and Stoorvogel, 2018). In summary, these findings reveal that EVs rely on specific environmental conditions to operate effectively.

### 2.3 EVs and intestinal barrier dysfunction in IBD

The gut must maintain tolerance to the constant foreign matter it encounters to stay balanced. Interestingly, intestinal permeability is a vital aspect in IBD etiology. The processes of intestinal barrier maintenance are altered in the inflamed gut, including downregulation of epithelial cadherin (E-cadherin), decreased mucus layer thickness, and malfunctioning of goblet and Paneth cells (Shen et al., 2022; Camilleri et al., 2012). EVs regulate epithelial cell permeability and tight junction integrity by transporting destructive or protective factors. In a healthy intestine, IECs enhance the repair of gut wall integrity by producing Annexin A1 (ANXA1)-containing EVs, an amino acid that promotes wound healing in patients (Leoni et al., 2015). The allylic hydrocarbon receptor (AHR) is an additional strategy in homeostasis regulation. Once stimulated, AHR produces IL-22, stimulates IL-10R levels, and reinforces gut epithelium’s tight junctions to preserve gut integrity (Stockinger et al., 2021). AHR also inhibits the expression of the IL-33 receptor, which is associated with the suppression of tumorigenicity 2 (ST2, strongly detected in IBD), while decreasing the synthesis of certain pro-inflammatory mediators, including IL-13. IL-13 stimulates STAT6 in epithelial cells and influences tight junctions in the intestinal epithelium (Imai et al., 2019; Chen Y. et al., 2021). IL-1β recruits granulocytes to the infection site, compromising the connectivity and integrity of gut epithelial cells (Coccia et al., 2012). IL-9 presence is also linked to changes in tight junction expression, with excessive IL-9 in the gut potentially compromising epithelial barrier integrity and tolerance to symbiotic bacteria, resulting in inflammation (Chen Y. et al., 2021). Moreover, TNF-α and IL-1β induce endoplasmic reticulum stress, affect Caco-2 cells (IECs), and significantly alter key proteins in the apical and basolateral membranes, including E-cadherin. This further disrupts the tight junctions of the intestinal epithelium (Chotikatum et al., 2018). Intestinal mucosa polymorphonuclear neutrophil (PMN) infiltration is common in IBD. Myeloperoxidase (MPO), which is released from PMNs, damages and compromises the gut barrier’s integrity. When escorted by EVs, MPO reaches the IECs, where it enhances inflammatory responses and hinders wound healing by regulating IEC migration and proliferation (Slater et al., 2017).

The control of the gut barrier is also significantly influenced by the ingesta-derived EVs. Table 1 shows how ingested EVs affect the gut barrier. In conclusion, it has been demonstrated that EVs from host cells significantly contribute to the pathophysiology of IBD by impairing the intestinal barrier and preventing the healing of intestinal wounds.

**TABLE 1:** The impact of ingesta-derived EVs on the gut barrier.

Effect on inflammation	Origin	Size	Major functional component(s)	Outcome	Reference (s)
Anti-inflammatory	Bovine milk	30.0–200.0 nm	Multiple proteins and miRNAs	Modulating the TLR4-NF-κB and NLRP3 signaling pathways helps regulate intestinal immune homeostasis, restore the balance between Treg and Th17 cells, and reshape the gut microbiota.	Tong et al. (2021)
	Grapefruit	105.7–396.1 nm	Naringenin	Increased HO-1 levels while lowering IL-1β and TNF-α production in colon macrophages.	Wang et al. (2014)
	Grape	343.0–418.0 nm	Multiple lipids, proteins, and miRNAs	Inducing intestinal stem cell proliferation and intestinal tissue remodeling.	Ju et al. (2013)
	Broccoli	18.3–118.2 nm	SFN	Activating AMPK to induce tolerogenic DCs.	Deng et al. (2017)
	Ginger	220–290 nm	Lipid, protein, miRNA, and ginger bioactive constituents (6-gingerol and 6-shogaol)	Preserving the gut barrier and replenishing the gut microbiota after being absorbed by IECs and macrophages.	Zhang et al. (2016)

EV, extracellular vesicle; miRNA, microRNA; TLR, Toll-like receptor; NF-κB, nuclear factor-κB; NLRP, Nod-like receptor pyrin domain-containing; Treg, regulatory T cell; Th17, T helper 17 cell; HO-1, heme oxygenase-1; IL-1β, interleukin-1β; TNF-ɑ, tumor necrosis factor-ɑ; SFN, sulforaphane; AMPK, adenosine monophosphate-activated protein kinase; IEC, intestinal epithelial cell.

### 2.4 EVs and the gut microbiota in IBD

The gut microbes definitely play an important role in intestinal inflammation (Lee and Chang, 2021). However, definitive cause–and–effect mechanistic relationships have been challenging to demonstrate outside specific animal models. Evidence from substantial experimental models indicates that although gut bacteria often drive immune activation, chronic inflammation in turn shapes the gut microbiota and exacerbates dysbiosis (Ni et al., 2017). IBD patients have a different gut microbiome than healthy individuals, as evidenced by downregulation of *Firmicutes* and upregulation of *Bacteroidetes*, *Actinobacteria*, and *Proteobacteria*, and an increase in the *Firmicutes-to-Bacteroides* ratio. There is also a notable decrease in intestinal flora diversity (Jacobs et al., 2016). Emerging evidence suggests that EVs derived from gut bacteria play a crucial role in the interaction between microbiota and host. For instance, *F. prausnitzii*, a member of the thick-walled phylum *F. prausnitzii* (Fp-Evs), raised the ratio of Tregs in the colon tissue of colitis mice and enhanced the protein expression of zona occludens (ZO)-1 and occludin. Fp-EVs downregulated the expression of the proinflammatory cytokines. Moreover, Fp-EV treatment markedly reduced the phosphorylation of several proteins, including nuclear factor-κB (NF-κB) and mitogen-activated protein kinase (MAPK), while also regulating the expression of nuclear factor erythroid 2-related factor (Nrf2) and heme oxygenase-1 (HO-1) (Ye et al., 2023). It is known that adherent-invasive *Escherichia coli* (AIEC), which is abundant in the gut mucosa of CD patients, impairs intestinal epithelial barrier stability and triggers the onset of gut fibrosis by modulating and destabilizing proteins at cell junctions, thereby increasing permeability. AIEC invasion increased the levels of IL-33 receptor ST2 in gut epithelial cells, which is necessary for the onset of gut fibrosis (Imai et al., 2019). The exosomes secreted by these infected IECs activate the host’s innate immune response and enhance intracellular replication of these pathogens. Abnormal bacterial colonization leads to inflammation by transporting microbial components via EVs. According to recent research, patients with intestinal barrier failure have a high concentration of lipopolysaccharide (LPS)-positive bacterial EVs (Tulkens et al., 2020). Besides LPS, their cargo also includes deoxyribonucleic acid (DNA), enzymes, RNA, peptidoglycan, and toxins. Their interaction with epithelial cells can regulate the gut barrier, control the proliferation and apoptosis of intestinal epithelial cells, and finally trigger the intestinal immune response (Chelakkot et al., 2018; Li et al., 2015; Kunsmann et al., 2015).

## 3 The role of extracellular vesicles in IBD-associated colorectal cancer

### 3.1 EVs as promoters of tumorigenesis in the inflamed gut

The function of EVs in tumor formation, survival, advancement, and other stages of tumorigenesis remains an active area of research. Numerous studies have shown that EV-mediated signaling can promote mutagenesis during the epithelial injury-repair cycles through genomic instability and epigenetic alterations. IBD-induced damage to intestinal epithelial cells leads to the release of EVs carrying mitochondrial DNA (mtDNA). These EVs are taken up by adjacent colonic epithelial cells (CECs), where the mitochondrial genome drives metabolic reprogramming, mitochondrial respiration, and reactive oxygen species (ROS) production, thereby promoting TGFβ1-mediated tumor progression (Guan et al., 2024). During the tissue repair phase, Wnt signaling molecules carried by EVs promote cell proliferation to repair the intestinal barrier. However, under inflammatory conditions, persistent Wnt signaling activation leads to abnormal expansion of the intestinal epithelial stem cell niche (Rahmati et al., 2024). The mitochondrial transcription factor A (TFAM) mRNA carried by MSC-EVs can alleviate mitochondrial damage and inflammation by stabilizing mtDNA (Zhao et al., 2021). Patients with gastrointestinal cancers have increased levels of certain circulating EV fractions compared to those with inflammatory gastrointestinal diseases such as IBD (Deutschmann et al., 2013; Mitsuhashi et al., 2016). This may be attributed to irregular EV secretion resulting from acidic conditions and hypoxia. Tumor pH levels between 6.0 and 6.8 suggest that increased acidity correlates with greater tumor aggressiveness. This acidic environment favors cancer cells that are resistant to treatment, which in turn secrete more EVs under stress (Fais et al., 2014).

### 3.2 EVs and the tumor microenvironment (TME) in IBD-associated CRC

**FIGURE 3:**
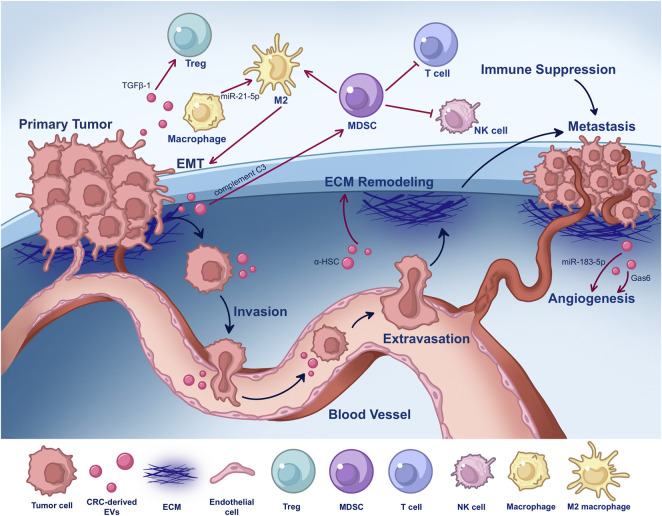
EVs derived from CRC alter the TME, promoting tumor growth and metastasis. CRC-derived EVs can polarize M2 macrophages and activate Treg cells, resulting in immunosuppression. Polarized M2 macrophages can, in turn, promote EMT. The production of MDSC suppresses the anti-tumor activity of T and NK cells while allowing tumor cells to proliferate. Tumor-derived EVs circulate to distant organs and modify the ECM, boosting tumor cell adhesion and encouraging angiogenesis. TGFβ-1, transforming growth factorβ-1; Treg, regulatory T cell; miR, microRNA; MDSC, myeloid-derived suppressor cell; NK cell, natural killer cell; EMT, epithelial-mesenchymal transition; ECM, extracellular matrix; α-HSC, activated hepatic stellate cell; Gas6, growth arrest specific 6.

The TME is diverse, comprised of extracellular matrix (ECM), stromal tissue, and malignant cells (Lei et al., 2020). CRC cells release EVs into the TME to communicate with immune cells and their own cells. It has been shown that EVs derived from CRC can alter the TME, leading to tumor development and spread by promoting immune evasion and a cancerous phenotype in recipient cells (Figure 3) (Glass and Coffey, 2022). A study has shown that CRC cells can secrete SEVs, which are absorbed by macrophages and contain miR-21-5p and miR-200a, thereby inducing M2-like polarization and PD-L1 expression. This results in increased abundance of PD-L1 CD206+ macrophages and decreased activity of CD8^+^ T cells in the CRC TME (Yin et al., 2022). During cancer treatment, persistent senescent tumor cells (STCs) generate abundant EVs that are rich in serine protease inhibitor 1 (SERPINE1). SERPINE1 binds to p65, facilitating its nuclear translocation and subsequently activating the NF-κB signaling pathway, which promotes the progression of recipient CRC cancer cells (Zhang D. et al., 2024). However, tumor exosomes can activate CD4+/CD25+/Foxp3+ Treg cells via TGFβ-1, leading to immunosuppression (Ciardiello et al., 2016). The production of myeloid-derived suppressor cells (MDSCs) can inhibit the antitumor functions of T cells and natural killer (NK) cells, thereby promoting tumor progression. In another study, Zhang et al. discovered that tumor-derived EVs transfer complement C3, which induces pulmonary macrophages to release CCL2 and CXCL1. This improves macrophage polarization and facilitates the recruitment of PMN-MSCs, eventually enabling tumor spread (Zhang Y. et al., 2025). Figure 3 shows how EVs derived from CRC alter the TME, promoting tumor growth and metastasis.

To sustain the survival and spread of rapidly growing malignant cells, angiogenesis is critical for supplying sufficient nutrition and oxygen (Huang et al., 2021a). Angiogenesis involves the formation of new blood vessels, which occurs when a vascular bud develops from an existing blood vessel or when a capillary wall germinates into the lumen of a blood vessel; then, columns form within this lumen. There is growing evidence that EVs are essential for angiogenesis (Wang et al., 2019; Shang et al., 2020). For instance, EVs derived from CRC perivascular cells expressed growth arrest-specific 6 (Gas6) and facilitated the recruitment of endothelial progenitor cells (EPCs) to tumors by activating the Axl pathway. This process leads to tumor revascularization after the withdrawal of antiangiogenic drugs (Huang et al., 2021b). Huang and colleagues observed that EVs from CRC cells were increased in Wnt4 in hypoxic settings. Wnt4 is a secreted protein signal from the Wnt family that plays a role in carcinogenesis (Li et al., 2014). These exosomes enriched with Wnt4 increased β-catenin nuclear translocation in endothelial cells in a hypoxia-inducible factor 1-α (HIF1α)-dependent way (Huang and Feng, 2017). However, Shang and the team discovered that CRC cell exosomes overexpressing miRNA (miR)-183-5p inhibited the proliferation, invasion, and tube formation ability of microvascular endothelial cells (HMEC-1) by upregulating forkhead box O1 (FOXO1), indicating that overexpressing miR-183-5p exosomes may be a useful therapeutic indicator for CRC (Figure 3) (Shang et al., 2020).

Tumor-derived EVs can affect tumor growth by promoting malignant cell proliferation and remodeling the ECM, where changes in composition, degradation, and stiffness are considered key contributors to tumor growth. Given their organic nutritional properties, tumor-derived EVs spread to distant organs and interact with and regulate the ECM complex, thereby increasing the adhesion of circulating tumor cells (CTCs) and paving the way for tumor cell infiltration (Kaushik et al., 2016). Since ECM dysregulation is often reported as the key step in the invasion-migration cascade, a comprehensive study of tumor cell EVs and their interactions with the ECM complex will help determine the drivers of distant organ activation and subsequent tumor invasion (Kaushik et al., 2016). An activated hepatic stellate cell (α-HSC) influences the growth and spread of CRC cells by modifying and augmenting the ECM (Figure 3) (Ahmad et al., 2003). Li and colleagues found through *in vitro* and *in vivo* experiments that the membrane glycoprotein dysadherin specifically acts on matrix metalloproteinase 9 (MMP9), enhances the invasiveness of CRC cells and the hydrolytic activity of ECM proteins, and activates cancer-associated fibroblasts (CAFs), thereby initiating ECM remodeling and amplifying cancer progression in the TME (Lee et al., 2024).

### 3.3 EVs and metastasis in IBD-associated CRC

The spread of tumor cells is a crucial factor in the progression of cancer. EVs are essential for EMT, creating a pre-metastatic milieu, and promoting CRC cell proliferation during metastasis (Rahmati et al., 2024). Metastases account for most of the deaths in CRC patients. The liver is the most prevalent location of metastasis, with around 30% of CRC patients acquiring hepatic metastases (Chen J. et al., 2020). This section mainly discusses the role of EV in liver metastasis of CRC.

The distinctive conversion of IECs to mesenchymal cells is known as EMT. Additionally, there is a shift towards a low-proliferation phase, and a breakdown of cell junctions and apical-basal polarity, which facilitates tumor cell movement and invasion (Pastushenko and Blanpain, 2019). After undergoing EMT, CRC cells release exosomes containing miR-106b-5p, which suppresses programmed cell death 4 (PDCD4) and activates the PI3Kγ/Akt/mTOR signaling pathway, thereby leading to M2 polarization. Conversely, M2 stem cells accelerate cancer cell motility and invasion by boosting the EMT process (Yang et al., 2021). Moreover, Liang and colleagues reported RNA pyrophosphohydrolase, a long non-coding (lnc) RNA, is significantly expressed in SW620 and HCT8 cells and physically interacts with β-III tubulin to promote malignant cell motility and EMT (Liang et al., 2019). The premetastatic niche has key features, including angiogenesis, vascular permeability, immunosuppression, reprogramming, lymphangiogenesis, organ tropism, and inflammation. These characteristics are essential for creating a favorable environment that allows malignant cells to settle and proliferate (Liu and Cao, 2016). There is evidence that upregulation of EV-mediated tissue inhibitor of matrix metalloproteinase-1 (TIMP1) in recipient fibroblasts induces ECM remodeling. However, HSP90AA, a heat-shock protein, can bind to EV-related TIMP1, thereby inhibiting TIMP1-mediated ECM remodeling, making EV-related TIMP1 a potential therapeutic target (Rao et al., 2022). Moreover, EV-associated miR-181a-5p metastasis in CRC cells with a high metastatic propensity stimulates TME remodeling, premetastatic niche development, and hepatic metastasis. This is accomplished by activating hematopoietic stem cells by inhibiting suppressor of cytokine signaling 3 (SOCS3) expression and enhancing the IL6/STAT3 pathway (Zhao S. et al., 2022).

CAFs are critical stromal cells that play major roles in tumor growth. CAFs secrete exosomes that promote CRC spread and chemotherapeutic resistance by increasing the cell’s stemness and EMT. For example, CAF cells activate the Wnt/β-catenin pathway by increasing the expression of miR-92a-3p exosomes and inhibit mitochondrial cell death by directly suppressing F-Box and WD Repeat Domain Containing 7 (FBXW7) and modulator of apoptosis 1 (MOAP1), thereby promoting cell stemness and EMT in CRC (Hu et al., 2019). EVs containing integrin beta-like 1 (ITGBL1) activate fibroblasts in distant organs by inducing the production of pro-inflammatory cytokines such as IL-8 and IL-6, ultimately promoting the growth of metastatic tumor cells (Ji et al., 2020). Furthermore, although cancer stem cells (CSCs) represent only a small portion of tumor cells, they are widely regarded as key drivers of tumorigenesis and play essential roles in shaping the tumor microenvironment and promoting metastasis. EVs derived from CSCs reportedly mediate cell crosstalk via the horizontal transfer of tumorigenic factors, such as oncogenes and proteins (van Niel et al., 2018). Studies have demonstrated that overexpression of miR-200c in CSCs leads to the release of EVs that carry excess miR-200c, thereby enhancing metastatic potential by promoting proliferation and inhibiting apoptosis (Tang et al., 2020). These results show how EVs derived from the TME affect recipient cells and play a crucial role in metastasis.

### 3.4 EVs as mediators of therapy resistance in IBD-associated CRC

Current chemotherapy regimens include both single-agent therapy, primarily based on 5-fluorouracil (5-FU), and multiple-agent combination medications such as oxaliplatin (OX), irinotecan, and capecitabine. Currently, the treatment of CRC still faces several challenges, such as low bioavailability of anticancer medications in the rectum and distal colon, the existence of efflux pumps in cancer cells, and the potential for medication exposure to healthy cells, which leads to adverse consequences and poor prognosis (Singh et al., 2022). Exosomes can influence drug resistance by increasing the number of efflux pumps in sensitive cancer cells. For example, overexpression of ATP-binding cassette (ABC) transporters, including ABCB1 (P-glycoprotein) and ABCC1 (multidrug resistance-associated protein), increases the release of 5-FU from malignant cells, reducing its intracellular concentration and effectiveness (Rahmanian et al., 2021). Notably, the most studied exosome-mediated mechanism of drug resistance involves the transfer of bioactive cargo via exosomes, with studies focusing on miRNA. A study attributed metastatic capacity, EMT, and 5-FU/OX resistance to CAF-derived exosomal miRNAs, including miR-92a-3p (Hu et al., 2019). Another study discovered that miR-196b-5p increases stemness and pharmacologic resistance to 5-FU in CRC cells by targeting the negative regulators of the STAT3 signaling pathway, SOCS1 and SOCS3 (Ren et al., 2017).

The involvement of EVs in drug resistance is a new research domain. Therefore, EV-mediated cargo selective delivery to cancer cells is an effective strategy to improve the therapeutic index and overcome drug resistance. For instance, sorafenib overcomes irinotecan resistance in CRC by increasing ibrutinib’s toxicity and promoting intracellular accumulation by blocking the drug-excreting pump ABCG2 (Mazard et al., 2013). EVs can also enhance targeted drug delivery, regulate immunological responses, and interact with the TME to make malignant cells more susceptible to therapy. However, long non-coding RNA (lncRNA, LINC01915) limits the use of EVs derived from CRC by normal fibroblasts (NFs) through the miR-92a-3p/Krüppel-like factor 4/cholesterol 25-hydroxylase (miR-92a-3p/KLF4/CH25H) pathway. This mechanism prevents angiogenesis and the transformation of NFs into CAFs, thereby inhibiting tumor growth (Zhou et al., 2021). In another study, CRC cells expressing IL-33 showed increased 5-FU sensitivity when T cells were present, thereby activating intrinsic apoptosis and signals associated with immunological destruction of tumor cells (Song et al., 2023). In TME, higher levels of milk fat globule-epidermal growth factor 8 (MFGE8) promote efferocytosis by stimulating macrophages to remove cisplatin-induced apoptotic cells, contributing to the lethal consequences of chemotherapeutic resistance (Ma et al., 2024). These findings shed fresh light on the potential synergistic use of chemotherapeutic drugs, EV inhibitors, and cell proliferation inhibitors in CRC therapy.

## 4 Novel inflammatory mediators: their interaction with extracellular vesicles in IBD and CRC

### 4.1 Specific examples of novel inflammatory mediators and their EV-mediated transport and regulation

**FIGURE 4:**
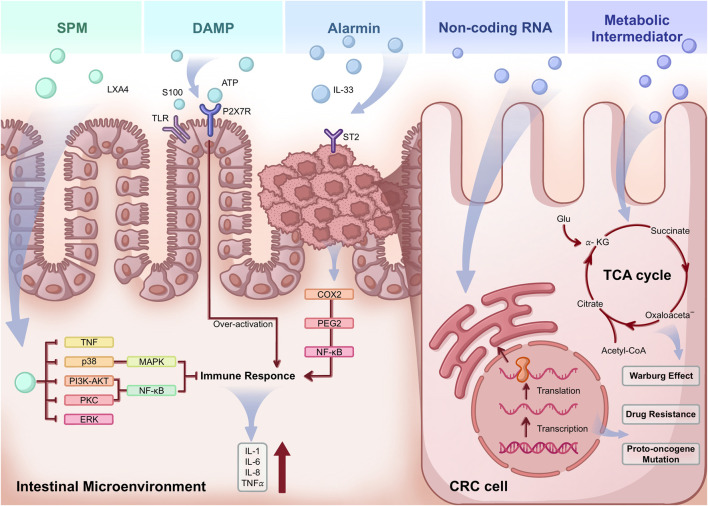
Illustrations of the role of new inflammatory mediators in the progression of IBD-CRC. LXA4, as an SPM, protects the immune balance of the intestine through multiple signaling pathways. ATP, as an extracellular DAMP, can combine with the overactivated P2X7 receptor, TLR, and other receptors to strengthen the inflammatory response and promote cancer. Alarmin usually plays paradoxical roles in IBD-CRC progression. Non-coding RNAs influence gene expression at numerous levels, resulting in mutations in proto-oncogenes, treatment resistance, and other variables that promote cancer cell malignancy. Metabolic intermediates, such as some amino acids, can complete the Warburg effect by affecting the TCA cycle. SPM, specialized pro-resolving mediator; DAMP, damage-associated molecular patterns; LXA4, Lipoxins A4; ATP, Adenosine triphosphate; IL, interleukin; TLR, Toll-like receptor; COX2, cycloxygenase-2; PEG2, prostaglandin E2; NF-κb, nuclear factor-κB; TNF, tumor necrosis factor; PI3K, phosphoinositide 3-kinase; AKT, protein kinase B; PKC, Protein kinase C; ERK, extracellular signal-regulated kinase; Glu, glucose; α-KG, alpha-ketoglutarate; CRC, colorectal cancer.

In IBD, novel inflammatory mediators activated by EVs, such as DAMPs, non-coding RNAs, metabolic intermediates, and specialized pro-resolving mediators (SPMs), are transmitted to epithelial cells and the TME to convey pro-tumor inflammatory signals, as evidenced by numerous specific examples (Figure 4).

#### 4.1.1 Specialized pro-resolving mediators

It is well recognized that the inflammatory response’s primary function is to shield the host from endogenous or exogenous threats. The inflammatory process is intricately designed to self-resolve to avert harm once dangerous stimuli have been eliminated. However, if inflammatory mechanisms are not effectively managed, they can cause tissue damage, as observed in chronic disorders like IBD. The inability to activate mechanisms that resolve inflammation, such as the removal of debris, may contribute to the onset of cancer (Fishbein et al., 2020). The beneficial function of the inflammatory response is ensured by the activation of resolution programs and the biosynthesis of proresolving mediators that counteract the action of inflammation-initiating mediators (Colgan, 2015). Among the effectors of resolution, an important role is emerging for specialized pro-resolving lipid mediators (SPMs): they govern resolution of inflammation via specific G-protein-coupled receptors and promote tissue healing and regeneration. SPM refers to a class of chemically and functionally distinct lipid mediators produced from polyunsaturated fatty acids. To address inflammation without suppressing the immune system, SPMs limit excess neutrophil infiltration, encourage the sequestration of cytokines that cause inflammation, and enhance macrophage efferocytosis (Jordan et al., 2021). Thus, SPM is regarded as a valuable immunoresolvent and a substitute for nonsteroidal anti-inflammatory drugs in the treatment of inflammatory diseases. Additionally, resolvin D1 in CAC suppresses c-Myc and TNF-α in CRC cells (Zhong et al., 2018). In IBD models, protectin D1n-3DPA and resolvin D5n-3DPA reduced leukocyte adhesion to TNF-α-activated human endothelial monolayers, thereby preventing colitis and intestinal injury (Gobbetti et al., 2017). Moreover, lipoxins (LXs), such as LXA_4_, are among the SPMs that have been widely studied (Figure 4). LXA_4_ inhibits the infiltration of CD45^+^ leukocytes and CD68^+^ macrophages in colorectal tumor tissues. LXA_4_ suppresses the expression of extracellular signal-regulated kinase, p38, and NF-κB in xenografts, inhibiting the growth and movement of CRC cells stimulated by activated macrophages in the conditioned medium (Liu et al., 2019). Although current research lacks evidence linking EVs and SPM in resolving intestinal inflammation, we can hypothesize that encapsulating SPM within EVs for targeted delivery to critical sites may modulate inflammatory pathways. The therapeutic potential of EV-mediated SPM in IBD and CRC represents a promising frontier worthy of exploration.

#### 4.1.2 DAMPs

DAMPs are chemicals that cells naturally create when they are under stress. High tumor apoptosis in malignancies causes stress and inflammatory signals that cause DAMPs to be secreted, which causes cancer cells to die immunogenically (Abu et al., 2021). However, because malignancies are highly adaptable, the lack of DAMPs to execute a complete and efficient anti-tumor response might result in opposing processes in which cancers exploit DAMPs to boost malignant growth and survival (Balkwill and Mantovani, 2001). Thus, it has been demonstrated that the release of DAMP in some cancer treatments or cell death causes an inflammatory response and may hasten the growth of tumors. According to certain research, EVs can emit DAMP, which then binds to immunological receptors such as the P2X7 receptor (P2X7R) and Toll-like receptors (TLRs). One significant extracellular DAMP, ATP, stimulates P2X7R, which functions as an alarm sensor in immunological cells and participates in other biological processes (Cheng et al., 2021). P2X7 is highly expressed in the intestinal mucosa of IBD patients. Prolonged stimulation of the P2X7R contributes to persistent inflammation and malignant alterations in the bowel. Conversely, inhibiting P2X7 reduces the occurrence of chemically induced experimental colitis (Figure 4) (Marques et al., 2014). Also, a class of proteins known to bind calcium and control both extracellular and intracellular activities, the S100 family has long been regarded as a DAMP due to its capacity to trigger inflammatory responses (Kligman and Hilt, 1988). In cancer, it has been reported that the S100 protein can be present in EVs and participate in carcinogenic effects (Wang et al., 2023). For instance, S100A10 significantly enhances the progression of hepatocellular carcinoma by transferring into EVs and regulating their protein payloads (Wang et al., 2023). Also, S100A5 and S100A7A proteins have been found in CRC cell lines (Hong et al., 2009; Tauro et al., 2013). In general, free S100 protein can act as a DAMP by binding to receptors such as the receptor for advanced glycation end products (RAGE) or TLR (Figure 4). TLR is a key molecule involved in inflammation-driven carcinogenesis, and overactivation of TLR leads to the progression of inflammatory diseases as well as tumor progression and metastasis (Naqvi et al., 2018). But research on the role of EV-mediated S100 proteins in CRC is unexplored, and its influence on inflammatory pathways is still uncertain. Since DAMP can affect tumor and pro-tumor activity, it is critical to comprehend the molecular processes that underpin its effects.

#### 4.1.3 Alarmins

Alarmin proteins appear to play paradoxical roles in the progression of inflammation and tumors, a contradictory role that has intrigued researchers for a long time. The alarmin proteins we primarily examine here are IL-33 and high mobility group box 1 (HMGB1).

IL-33 is a member of the IL-1 family and is an alarmin protein cytokine that plays a vital role in tissue homeostasis and repair, as well as cancer. In recent years, numerous studies have demonstrated IL-33’s multifunctional role in regulating Th2 immune responses (Liew et al., 2010), boosting anti-tumor CD8^+^ T and Th1 cell immunity (Gao et al., 2013), and stimulating and attracting APCs (Rank et al., 2009), as well as its versatile cytokine activity across various malignancies (Gambardella et al., 2024). Furthermore, IL-33 has been discovered to be intimately connected with tumor cell proliferation, stemness, apoptosis, migration, or chemotherapy resistance, and tumor progression in several malignancies, including colon, ovarian, and breast cancer (Tong et al., 2016; Fang et al., 2017; Kim et al., 2015). A study found that IL-33 acts through its receptor ST2, increasing cyclooxygenase-2 (COX2) expression via NF-κB signaling. This suggests that IL-33 supports the proliferation of CRC that relies on COX2/prostaglandin E2 (PGE2) (Li et al., 2018). Moreover, a soluble form of the IL-33 receptor (sST2) modulates the TME by stimulating blood vessel development, Th2 and Th1 reactions, macrophage invasion, and M2a polarization, thereby adversely impacting CRC metastasis and confirming the pro-tumor effect of IL-33 in reverse (Figure 4) (Akimoto et al., 2016).

HMGB1 can also be translocated to the extracellular environment, acting as a warning signal to trigger immunological responses and participate in the control of inflammation and the advancement of cancer. Its effects on tumors include initiating inflammation, enhancing cell growth, promoting infiltration and metastasis, driving angiogenesis, contributing to chemical resistance, and supporting anti-tumor immunity (Abu et al., 2021). In CRC patients, HMGB1 serum levels are significantly higher than in IBD patients and healthy individuals (Wang and Huang, 2025). Research indicates that as solid tumors grow in hypoxic conditions, necrotic cells release HMGB1 into the extracellular environment. This extracellular HMGB1 activates the MAPK and myeloid differentiation primary response 88 (MyD88)-dependent NF-κB pathway, which subsequently stimulates the release of cytokines such as IL-6 and IL-8. These cytokines ultimately promote tumor proliferation and metastasis (Yuan et al., 2020). In the acidic TME, macrophages are activated to an M2 phenotype and secrete more HMGB1, which promotes the malignancy of CRC cells by stimulating Wnt, ERK, and EMT signaling pathways (Gao et al., 2023).

#### 4.1.4 Non-coding RNAs

Non-coding RNA (ncRNA) refers to RNA molecules that do not encode proteins. This group includes miRNAs, lncRNAs, and circular RNAs (circRNAs). ncRNA can influence gene expression at various levels by interacting with DNA, RNA, or proteins. This includes modifying chromosomes, regulating gene transcription, affecting mRNA translation, and influencing protein biological functions, which can enhance cell proliferation due to their oncopreventive characteristics (Figure 4) (Statello et al., 2021). In the previous article, we have repeatedly mentioned the role of ncRNA in IBD and related CRC progression (Liang et al., 2019; Hu et al., 2019; Zhou et al., 2021).

During carcinogenesis, accumulating epigenetic and genetic changes typically silence tumor suppressor genes and promote the production of oncogenes. It is well understood in CRC that various proto-oncogene alterations affecting *KRas, BRaf, PTEN, PIK3CA,* or *TP53* increase CRC cell proliferation by disrupting essential cell cycle players (Berg et al., 2010). For example, CRC cell-derived EV miRNAs promote tumor progression by inhibiting TP53 expression in fibroblasts (Yoshii et al., 2019). This is also suitable for EVs rich in DeltaNp73, promoting the carcinogenic potential of recipient cells (Soldevilla et al., 2014). In a recent study, mutated KRAS and BRAF DNA were found in exosomes extracted from the plasma of CRC patients whose tumors carried these mutations (Galbiati et al., 2021). The KRAS protein can also influence miRNA sorting into exosomes, with miRNA-100 being preferentially packaged and released in exosomes from mutant KRAS CRC cells (Hinger et al., 2018). LncRNAs are linear transcripts longer than 200 base pairs that regulate gene expression but do not encode proteins. The potential role of numerous lncRNAs in the development and progression of CRC has been explored (Chen L. J. et al., 2023). Deng et al. found that colorectal cancer-associated lncRNA (CCAL) increased CRC cells’ resistance to oxaliplatin. Functional investigations revealed that CCAL is transported from CAFs to CRC cells via exosomes, stimulating β-catenin signaling, triggering chemoresistance, and lowering apoptosis (Deng et al., 2020). Additionally, circular RNA (circRNA) is a single-stranded RNA that forms a closed loop and has gene-regulatory functions. ciRs-7 has been identified as both a marker and a therapeutic target in CRC. It acts as a sponge for tumor-suppressive miRNAs, which activate the epidermal growth factor receptor (EGFR) and are associated with poor patient survival (Weng et al., 2017). The specific roles of EVs and their assembled ncRNAs in CRC progression are shown in Table 2.

**TABLE 2:** The specific role of EV and its assembled ncRNA in CRC progression.

EV ncRNA	Target	Origin	Expression pattern	Major mechanism	Reference(s)
MiR-21	PDCD4	CRC cell	Increased	Induces resistance to 5-FU therapy by downregulating PDCD4 and promotes CRC cell proliferation by activating ECM mediators	Sun et al. (2020)
Let-7a miRNA	SNAP23	CRC cell	Increased	Inhibits the secretion of EVs that directly target SNAP23 and also suppresses OXPHOS via SNAP23, thereby inhibiting the progression of CRC.	Liu et al. (2021)
MiR-425-5p	PTEN	CRC cell	Increased	Modulates the PI3K/AKT/mTOR signaling pathway to promote CRC progression.	Lenart et al. (2024)
MiR-34a-5p	c-MYC	MSC	Decreased	Regulates c-MYC-DNMT3a binding and epigenetic modifications to modulate PTEN and inhibit CRC development.	Zhao et al. (2022b)
MiR-185-5p	TEAD1	CRC cell	Decreased	It prevents cancer by inhibiting the Wnt/β-catenin pathway.	Dong-Xu et al. (2015), Yu et al. (2022), Shi et al. (2023)
MiR-143-3p	*KRAS* and *DNMT3A* genes	CRC cell	Decreased	Directly prevents translation of the *DNMT3A* and *KRAS* genes, slowing tumor cell growth.	Lenart et al. (2024), Ahadi (2020)
LncRNA ASB16-AS1	miR-185-5p	CRC cell	Increased	Regulates miR-185-5p to modulate TEAD1-Hippo signaling, which promotes CRC progression.	Yu et al. (2022)
LncRNA DANCR	miR-185-5p	CRC cell	Increased	Suppresses miR-185-5p and indirectly upregulates HMGA2 expression, boosting CRC cell proliferation and migration.	Lu et al. (2022)
LncRNA CPS1-IT1	HIF-1α	CRC cell	Decreased	Inhibits hypoxia-induced autophagy by deactivating HIF-1α, thereby suppressing metastasis and EMT.	Zhang et al. (2018)
LncRNA SNHG12	TGF-β	CRC cell	Increased	Activates the TGF-β/Smad2/3 signaling pathway to promote EMT and CRC metastasis.	Zhao et al. (2025)
LncRNA HOTTIP	miR-214	CRC cell	Increased	Downregulate miR-214 to increase KPNA3 expression and improve mitomycin resistance.	Chen et al. (2020b)
LncRNA XIST	ZEB1	CRC cell	Increased	Modulates ZEB1 expression via competitive miR-200b-3p to control CRC growth and metastasis.	Chen et al. (2017)
cricN4BP2L2	EIF4A3	CAF	Increased	Increases CRC cells’ stemness and OXA resistance by upregulating EIF4A3.	Qu et al. (2022)
circTAX1BP1	VIRMA	CAF	Increased	Binds to VIRMA to trigger downstream signaling pathways that improve paracrine TGF-β and EMT.	Tan et al. (2025)
circATG4B	TMED10	CRC cell	Increased	Competitively binds to TMED10 through its encoded protein circATG4B-222aa, promoting autophagy and enhancing resistance to oxidative stress.	Pan et al. (2022)
circTUBGCP4	miR-146b-3p	CRC cell	Increased	Upregulates PDK2 via sponge miR-146b-3p, activating Akt signaling and inducing endothelial cell polarization, which enhances angiogenesis and metastasis.	Chen et al. (2023c)
circVCP	miR-9-5p	CRC cell	Increased	Modifies the miR-9-5p/NRP1 axis to increase cell motility, invasion, and proliferation, and subsequently, activates M2 polarization in macrophages and inhibits M1 polarization.	Tang et al. (2023)
circCOL1A2	miR-665	CRC cell	Increased	Sponge miR-665 enhances LASP1 expression and regulates CRC phenotype, thereby accelerating CRC progression.	Miao et al. (2023)

EV, extracellular vesicle; ncRNA, non-coding RNA; CRC, colorectal cancer; miR, microRNA; PDCD4, programmed cell death 4; 5-FU, 5-fluorouracil; ECM, extracellular matrix; SNAP23, synaptosome-associated protein 23; OXPHOS, oxidative phosphorylation; PTEN, phosphatase and tensin homolog deleted on chromosome 10; PI3K, phosphoinositide 3-kinase; AKT, protein kinase B; mTOR, mechanistic target of rapamycin; c-MYC, myelocytomatosis viral oncogene homolog; MSC, mesenchymal stem cell; DNMT3a, DNA methyltransferase 3a; lncRNA, longnon-coding RNA; TEAD1, TEA domain transcription factor1; ASB16-AS1, ASB16 antisense RNA 1; DANCR, differentiation antagonizing non-coding RNA; HMGA2, high mobility group protein A2; CPS1-IT1, CPS1 intronic transcript 1; HIF-1α, hypoxia-inducible factor-1 alpha; EMT, epithelial-mesenchymal transition; SNHG12, small nucleolar RNA host gene 12; TGF-β, transforming growth factor-β; Smad, small mother against decapentaplegic; HOTTIP, HOXA distal transcript antisense RNA; KPNA3, Karyopherin subunit alpha 3; XIST, X inactive-specific transcript; ZEB1, zinc-finger E-box-binding homeobox 1; N4BP2L2, NEDD4 binding protein 2 like 2; EIF4A3, Eukaryotic initiation factor 4A-III; CAF, cancer-associated fibroblast; OXA, Oxaliplatin; TAX1BP1, Tax 1 binding protein 1; VIRMA, Virilizer-like m6A methyltransferase-associated protein; ATG4B, autophagy-related gene 4B; TMED10, transmembrane p24 trafficking protein; TUBGCP4, tubulin-gamma complex-associated protein 4; PDK2, pyruvate dehydrogenase kinase 2; VCP, valosin-containing protein; NRP1, neuropilin-1; COL1A2, collagen type I alpha 2 chain; LASP1, LIM and SH3 protein 1.

#### 4.1.5 Metabolic intermediates

The least researched part of EV is the metabolic group, a collection of chemicals most closely associated with cell phenotype. Nonetheless, the metabolome of migrating EVs in human blood and other bodily fluids is particularly fascinating, as it may aid in diagnosing cancer and various illnesses. Also, EV metabolomes can provide valuable insights into intercellular communication. Organic acids, nucleotides, sugars and their derivatives, carnitines, vitamins and related metabolites, and amines are commonly found in EVs (Zebrowska et al., 2019). Even small metabolic perturbations can promote reprogramming of cancer cell metabolism, presenting the role of EVs as key in this process (Yang et al., 2020).

Tumor cell growth constantly requires the maximum nutritional capacity to meet increased biosynthetic and bioenergetic requirements. Therefore, throughout the natural course of carcinoma, malignant cells must maintain high metabolic flexibility to adapt to continual changes in the tumor and its environment. In an EV, it may symbolize the parent cell transferring a milieu to the recipient cell, thereby modifying the recipient cell’s metabolism. A study has shown that EVs derived from visceral adipose tissue (VAT) in obese patients are enriched in glycolytic enzymes, such as triose phosphate isomerase (TPI1). This enrichment was associated with increased TPI1 protein levels in CRC cells and elevated glycolytic activity (Haque et al., 2025). Amino acids transported by EVs, such as glutamine and leucine, have been shown to significantly influence the tricarboxylic acid (TCA) cycle in recipient-malignant cells, thus improving the nutritional status of rapidly growing and dividing cells (Figure 4) (Zhao et al., 2016). For example, succinate, together with three other oncometabolites (D-2-hydroxyglutarate, L-2-hydroxyglutarate, and fumarate), is a small molecule that accumulates in cancer cells formed by gain-of-function or loss-of-function mutations in genes encoding enzymes of energy metabolism pathways. All four oncometabolites are formed in the mitochondria (tricarboxylic acid cycle) and can cause similar changes in cancer cells, such as hypermethylation and pseudohypoxia (Dando et al., 2019). One study demonstrated that the metabolite succinate was upregulated 1.86- to 2.88-fold (log2) in EVs derived from all cancer cell lines compared with the control group (Palviainen et al., 2020). TME can also promote cancer progression by inducing fatty acid beta-oxidation (FAO) in cancer cells through the uptake of free fatty acids (FFAs) via CD36, which are released by cancer-associated adipocytes (Pascual et al., 2017). Tumor angiogenesis is mediated by highly glycolytic TME-associated endothelial cells (Rohlenova et al., 2018). In addition, metabolism largely polarizes tumor-associated macrophages (TAMs) into M1 or M2 phenotypes. M1 cells are mainly glycolytic, whereas M2 cells predominantly depend on FAO and oxidative phosphorylation (OXPHOS) (Zhu et al., 2015). Therefore, these TME-derived cells can release EVs. These subsequently affect the metabolism of cancer cells, aiding in their growth.

#### 4.1.6 Evidence-based prioritization of EV-associated mediators in the IBD-to-CRC transition

Although numerous EV-associated inflammatory mediators contribute to the progression from chronic intestinal inflammation to CRC, the robustness of mechanistic and translational evidence differs markedly among mediator classes. Current experimental and clinical data indicate that EV-associated non-coding RNAs, particularly miRNAs and lncRNAs, exhibit the strongest evidence for driving the IBD-to-CRC transition. Tumor and stromal cell-derived EV miRNAs have been shown to directly regulate oncogenic pathways. For example, EV-associated miR-99a-5p drives fibroblast reprogramming into cancer-associated fibroblasts via NF-κB signaling to promote metastasis (Zhou et al., 2026), miR-143-3p from M2 macrophage EVs facilitates CRC progression through ZC3H12A/C/EBPβ (Zhou et al., 2023), and EV-contained miR-200 family members regulate fibroblast-to-myofibroblast differentiation relevant to stromal remodeling (Bhome et al., 2022); similarly, macrophage-derived exosomes carrying lncRNA NEAT1 enhance IBD-associated CRC development and stemness via the miR-34a-5p/PEA15 axis (Liu et al., 2025). Moreover, the detection of mutated KRAS and BRAF DNA in circulating exosomes from CRC patients underscores the translational relevance of EV cargo by linking it to key driver mutations in colorectal tumorigenesis (Hao et al., 2017; Martinez-Cannon et al., 2021; Nouri et al., 2025). Collectively, EV-associated ncRNAs and oncogenic nucleic acids currently represent the most substantiated mediators connecting chronic inflammation to malignant progression.

A secondary level of evidence supports the involvement of EV-associated DAMPs and alarmins, notably HMGB1 and ATP-P2X7R axis, which are well-documented in sustaining chronic inflammation, activating NF-κB, and TLR2/4 signaling pathway, and remodeling the tumor microenvironment (Foelsch et al., 2024; Chen et al., 2016; Neves et al., 2014; Wan et al., 2016). However, although these mediators strongly contribute to inflammation-driven carcinogenesis, direct evidence for EV-mediated transfer as a causal mechanism in the IBD-to-CRC transition remains limited. Similarly, IL-33 shows clear pro-tumorigenic activity in CRC via COX2/PGE2 and NF-κB signaling, yet its EV-mediated delivery in this context requires further investigation (Li et al., 2018; Katz-Kiriakos et al., 2021).

In contrast, EV-associated metabolic intermediates and SPMs represent emerging areas of research. Tumor-derived EV metabolites, such as succinate and glycolytic enzymes, influence metabolic reprogramming and the tumor microenvironment, but mechanistic data linking EV-mediated metabolic transfer to inflammation-driven colorectal carcinogenesis are still developing (Haque et al., 2025; Palviainen et al., 2020). Likewise, while SPMs exhibit anti-inflammatory and anti-tumor effects in experimental models, evidence for their EV-mediated delivery in IBD or CRC remains largely speculative.

Overall, current evidence positions EV-associated ncRNAs and oncogenic nucleic acids as the most compelling mechanistic and translational mediators in the IBD-to-CRC transition, followed by DAMPs and alarmins with significant but less EV-specific validation, whereas EV-mediated lipid mediators and metabolic intermediates represent promising yet comparatively underexplored areas for future study.

### 4.2 Crosstalk and synergistic effects

A tumor needs a sophisticated network of cell communication, much like any multicellular organism. In complex tissue environments, bidirectional interference is necessary for metastatic development, migration, multi-barrier invasion, effective colonization, and sustained growth. Aside from EVs’ potential to alter our understanding of cancer cell-stromal cell interaction in TME, their ability to selectively deliver diverse cargos to recipient cells has generated optimism (Patras and Banciu, 2019). EVs, as key intercellular communication, play a “bridge” role in the malignant transformation of IBD to CRC by carrying proteins, lipids, nucleic acids, or other novel inflammatory mediators. EVs can transport and regulate multiple inflammatory mediators simultaneously, driving tumor development in IBD-related chronic inflammation. More importantly, different EV cargoes have significant synergistic effects in driving inflammation and tumor development. In addition to transmitting oncogenic activity among malignant cell subsets, it has been shown that horizontal transfer of oncogenic, constitutively active EGFRvIII via EVs can activate autocrine vascular endothelial growth factor (VEGF) signaling in endothelial cells, thereby promoting tumor angiogenesis (Al-Nedawi et al., 2008; Al-Nedawi et al., 2009). On the other hand, malignant cells can alter and recycle fibroblast-secreted vesicles from the ECM. This process activates autocrine Wnt-planar cell polarity, increasing tumor cell motility and metastasis (Luga et al., 2012).

## 5 Therapeutic potential of targeting EVs and novel inflammatory mediators in IBD and CRC

### 5.1 EV-based diagnostics and biomarkers

Numerous studies have shown the benefits of EV-dependent diagnosis. EVs function as biomarkers for a variety of diseases, improving targeting capabilities compared to conventional histopathological diagnostics. They offer richer biological information, are collected with less invasiveness, and are more appealing to patients, particularly in the context of malignancies and inflammatory diseases (Console et al., 2019). Presently, research is increasingly focused on using EVs derived from biofluids, such as plasma, serum, stool, and tissue, as well as bowel lavage fluid, for the diagnosis and monitoring of IBD and CRC (Alorda-Clara et al., 2023). The identification of specific EV cargoes, such as novel inflammatory mediators or ncRNAs, can serve as biomarkers for disease activity, prognosis, and therapeutic response. Currently, numerous biomarkers are widely used in clinical practice, including serum C-reactive protein levels, *NOD2* gene expression, and inflammatory cytokines. However, none of these markers can be used alone for an accurate diagnosis of IBD because they are vague (Shen et al., 2022). Therefore, it is crucial to establish a precise and convenient EV-dependent diagnostic system for obtaining samples from IBD patients’ serum, lumen contents, or stool. According to the Reporting Recommendations for Tumor Marker Prognostic Studies (REMARK) guidelines, Rao et al. demonstrated that EV-bound TIMP1 serves as a powerful circulating biomarker for noninvasive preoperative risk assessment in individuals with colorectal liver metastases. Their findings further show that measuring EV-related TIMP1 is preferable to detecting soluble TIMP1 in total serum for noninvasive diagnosis. In this regard, assessing preoperative levels of EV-bound TIMP1 could serve as a complementary tool for molecular risk assessment and individualized treatment planning (Rao et al., 2022). Moreover, these data support the hypothesis that enriched EV in total serum can increase the production of tumor-specific protein biomarkers. As a result, this EV enrichment may enhance the diagnostic potential of liquid biopsy biomarkers and reduce false-positive rates in healthy individuals. ANXA1 is a proresolving regulator essential for epithelial wound healing, functioning through Rac2-dependent nicotinamide adenine dinucleotide phosphate oxidase-1 and acting as an inflammatory inhibitor. This protein is calcium-dependent and binds to phospholipids. During inflammation, such as in active IBD, IECs package ANXA1 into EVs, which are then distributed throughout the body, elevating serum levels. ANXA1-simulating peptides encapsulated in targeted polymer nanoparticles accelerated wound healing in the colon and recovery from experimental colitis in mice (Leoni et al., 2015). Thus, elevated ANXA1 levels in IEC-derived EVs could be a unique IBD diagnostic approach. Also, EVs containing nucleotides may serve as biomarkers for CRC. A 2015 study by Polytarchou et al. demonstrated that miR-214 is associated with the progression of IBD and that reducing its expression can slow the development of colitis and CRC in mice (Polytarchou et al., 2015). Serum exosomes expressing miR-320d are a promising noninvasive diagnostic biomarker that can discriminate between metastatic and non-metastatic CRC (Tang et al., 2019).

### 5.2 EV-based therapeutics

Over the past decade, efforts have focused on developing nanoparticle-based medication delivery technologies. Nevertheless, during the circulation cycle, the mononuclear phagocyte system removes nanoparticles. In contrast, EVs are naturally derived from cells and the microbiota, thus exhibiting superior biocompatibility and stability (Chang et al., 2020). Exosomes can be modified by parental cells to have the necessary targeting capacity and can carry a range of therapeutic substances. Exosomes can integrate the benefits of existing medication carriers that utilize synthetic nanoparticles with the cell-mediated delivery method while overcoming the drawbacks of both approaches. For instance, growing evidence indicates that MSCs secrete a variety of bioactive mediators capable of modulating immune and inflammatory responses, as well as promoting intestinal tissue regeneration. However, MSC therapy remains largely in the experimental phase due to significant limitations such as safety concerns, insufficient cell survival rates, immune rejection, and high costs. In contrast, EVs derived from MSCs exhibit biophysical properties highly similar to those of MSCs themselves and are considered potentially safer and more effective than MSCs (Zhou et al., 2019). Therefore, SEV derived from MSCs has been considered as a good carrier for drug delivery. Exosomes from MSCs transport miRNAs, cytokines, and other cargoes to the immunological milieu, where they influence immune effector cell function. A study highlights that the chemotherapeutic paclitaxel (PTX) can be directly incorporated into MSC cultures to generate SEVs with high PTX-dependent activity, which significantly suppresses cancer cell proliferation (Pascucci et al., 2014). Nonetheless, due to the potential PTX metallization during cell processing, drug loading efficiency may be limited (Pascucci et al., 2014). MSCs-EVs are also considered a promising new therapeutic approach for treating inflammatory diseases and cancer. MSC therapy for colitis focuses on inhibiting colonic macrophages, reducing pro-inflammatory cytokine levels, and inhibiting the NF-κB pathway, particularly following inflammation (Yang et al., 2015).

Extensive research has been done on the use of EV-like nanoparticles to treat IBD. Wang and the team found that grapefruit produced exosome-like vesicles that were specifically absorbed by intestinal macrophages. These vesicles increased heme oxygenase-1 levels and inhibited TNF-α and IL-1. As a result, they advanced the development of an EV-based oral drug delivery system for macrophage targeting (Wang et al., 2014). In 2018, Zahra and her team used gut organoids to deliver mesalamine nanoparticles for the treatment of IBD (Davoudi et al., 2018). In 2019, Han and colleagues enhanced the application of bioadhesive chitosan materials in colloidally stable nanotherapeutics. This exhibited safe and precise accumulation to local diseased lesions in the gastrointestinal tract (Han et al., 2019).

These findings show that EVs from cancer cells are effective carriers for delivering chemotherapeutic agents or nucleotides. However, given the tumor-promoting potential of EVs derived from malignant cells (Yoshii et al., 2019; Soldevilla et al., 2014), the safety and long-term effects of using EVs as drug-delivery vehicles must be thoroughly examined. Additional engineered EVs designed as drug-delivery vehicles for the treatment of IBD and CRC have been included, as summarized in Table 3.

**TABLE 3:** Engineered EVs as drug delivery vehicles to treat IBD and CRC.

Therapeutic strategies	Engineered carrier	Type of condition(s)	Consequences of action	Reference(s)
IL-27	MSC-derived EV encapsulated into DMA-modified hydrogel	IBD	Firmly adheres to the colon’s surface, lessens inflammation, and restores the compromised barrier.	Nie et al. (2023)
Rabex	Red cabbage-derived EV engineered by surface conjugation with hyaluronic acid	IBD	Efficiently targets and inhibits macrophage inflammation and promotes regeneration of colonic epithelial cells.	Kang et al. (2024)
MicroRNA 31 mimic	Peptosome nanoparticle derived from hydrolyzed alpha-lactalbumin, encapsulated into OKGM	IBD	Reduces the inflammatory response of the colon epithelium and regulates Wnt and Hippo signaling pathways to promote epithelial regeneration after injury.	Tian et al. (2019)
MicroRNA-146a mimic	LNP	IBD	Targets macrophages, downregulates inflammation-related genes, and prevents excessive production of pro-inflammatory cytokines.	Suri et al. (2025)
Magnolol	pH/redox dual-responsive butyrate-rich polymer nanoparticle	IBD	Prevents the synthesis of chemokines and pro-inflammatory cytokines, restores lipid metabolism, and modulates the gut microbiota.	Fan et al. (2024)
W-PB	Electrostatically crosslinked hydrogel composed of alginate and chitosan	IBD	Protects intestinal cells from oxidative injury by inhibiting *Escherichia coli* growth and neutralizing excess RONS.	Luo et al. (2025)
PPy and PFD	Probiotic inulin hydrogel	IBD	Inhibits fibroblast proliferation, reduces pro-inflammatory cytokine levels, enhances intestinal epithelial barrier repair, and inhibits intestinal fibrosis.	Wu et al. (2025)
PMX205	Eudragit S	IBD	Ensures effective drug delivery to the colon, mucosal adhesion, and sustained drug release to inhibit complement in the inflamed gut and restore homeostasis.	Cui et al. (2025)
siMDM2	Engineered EV derived from patient primary culture cells	CRC	Inhibits MDM2-mediated ubiquitination and degradation of NDUFB8, thereby reducing intracellular ROS production under chemotherapy stress and enhancing the efficacy of OXA-based chemotherapy.	Li et al. (2025)
siCCL24	Engineered EV derived from patient primary cells	CRC	Reduces CCL24 in TME and significantly enhances sensitivity to anti-angiogenic therapy.	Wang et al. (2025)
MTI and OXA	PLGA nanoparticles coated with a neutrophil membrane vesicle	CRC	Reverses the EMT induced by *Fusobacterium nucleatum *and reshapes the immunosuppressive microenvironment, thereby inhibiting the development of CRC and liver metastasis.	Niu et al. (2025)
Tumor antigen	OMV from *Escherichia coli* induced by monosaccharide arabinose	CRC	Passes through the intestinal epithelium into the lamina propria and stimulates the maturation of DCs.	Yue et al. (2022)
Erastin and FdUMP and siPD-L1	GE11 peptide-modified LNP containing a calcium phosphate core	CRC	Achieves effective ferroptosis and apoptosis in cells by coupling GSH depletion with ROS overexpression, while simultaneously downregulating PD-L1 on the cell surface to improve tumor-cell/CD8 T-cell identification.	Cao et al. (2024)
NMS-873 and P1C4	omPEG-coated SLN	CRC	Coordinates immunological reprogramming, produces ERS, reverses EMT/CSC, disrupts NETs, and inhibits immune checkpoints before efficiently overcoming resistance to chemotherapeutic immunotherapy.	Lo et al. (2025)
Curcumin and 5-FU	Silk fibroin	CRC	Effectively inhibits tumor cell proliferation and growth with low cytotoxicity.	Yuan et al. (2025)
Galunisertib	Gelatin-covered diatomite nanoparticles, encapsulated in an enteric matrix	CRC	Targets the L1-CAM on metastatic cells and continuously releases galunisertib under intestinal pH conditions.	Tramontano et al. (2023)
Doxorubicin	Polymeric nanoparticles composed of PEG and PLA, functionalized with the T7 peptide	CRC	Reduces systemic toxicity while precisely inducing CRC cell death by targeting TfR1.	Pina et al. (2025)
DTX and ATR	Lactoferrin nanoparticles	CRC	Protects drug payloads from degradation in the upper gastrointestinal tract, reduces burst drug release, and effectively treats CRC.	Elmorshedy et al. (2023)

EV, extracellular vesicle; IBD, inflammatory bowel disease; CRC, colorectal cancer; IL, interleukin; MSC, mesenchymal stem cell; DMA, dopamine methacrylamide; OKGM, oxidized konjac glucomannan microspheres; LNP, lipid nanoparticle; W-PB, multifunctional nanozyme; RONS, reactive oxygen and nitrogen species; PPy, polypyrrole nanozymes; PFD, antifibrotic drug pirfenidone; siMDM2, small interfering RNA (siRNA) targeting murine two‐microsomal homolog gene 2; NDUFB8, NADH, dehydrogenase ubiquinone 1beta subcomplex 8; ROS, reactive oxygen species; OXA, oxaliplatin; siCCL24, siRNA, targeting C-C motif chemokine ligand 24; TME, tumor microenvironment; MTI, metronidazole; PLGA, poly lactic-co-glycolic acid; EMT, epithelial-mesenchymal transition; OMV, outer membrane vesicle; FdUMP, 2'-deoxy-5-fluorouridine 5'-monophosphate sodium salt; siPD-L1, small interfering RNA, targeting PD-L1; GSH, glutathione; P1C4, bispecific PD-L1/CTLA-4, aptamers; omPEG, orthogonally masked polyethylene glycol; SLN, solid lipid nanoparticle; ERS, endoplasmic reticulum stress; CSC, cancer stem cell; NET, neutrophil extracellular trap; 5-FU, 5-fluorouracil; PEG, poly ethylene glycol; PLA, poly lactic acid; TfR1, Transferrin receptor 1; DTX, docetaxel; ATR, atorvastatin.

### 5.3 Targeting novel inflammatory mediators

A growing number of studies are targeting novel inflammatory mediators to develop therapeutic strategies that block their production, release, or signaling.

In terms of drug efficacy, it may be crucial to effectively load cancer treatment drugs into a given population of EVs. One effective method for loading malignant therapies into EVs is to incorporate drugs via donor cells, which contrasts sharply with the direct loading of therapeutic drugs into isolated EVs. In this approach, microRNAs are often delivered into sEVs via donor cells. Alexander and colleagues found that two key miRNAs involved in inflammation, endogenous miR-155 and miR-146a, are released from DCs in exosomes and subsequently absorbed by recipient DCs. Exogenous miRNA suppresses target genes and changes cell responses to endotoxins by increasing exosome-delivered miR-155 and lowering inflammatory gene expression via miR-146a (Alexander et al., 2015). miR-155 and miR-146a crosstalk with CAFs to increase CRC metastasis driven by CXCL12/CXCR7 (Wang et al., 2022).

Additionally, exosomes containing various antigenic peptides can modulate intestinal immunity by interacting with DC. Exosomes from bone marrow DCs modified by TGF-β1 inhibited the Th17 response and increased Tregs, preventing IBD development in mouse models. Mice’s drug-induced colitis is less severe when exosomes from DCs generated from bone marrow are treated with IL-10 (Heidari et al., 2021). Naqvi et al. employed nucleic acid-binding polymers, third-generation polyamidoamine dendrimers, to limit TLR activation by targeting and directly binding circulating EV DAMPs, thereby inhibiting tumor invasion and metastasis (Naqvi et al., 2018).

As we mentioned in the previous section, the carcinogenic effects of P2X7R make its antagonists potential targets for cancer therapy, such as oxidized ATP (oxATP), KN-62, A740003, and A438079 (Drill et al., 2021). However, concerns have been raised about the use of P2X7R-targeting biologics as anticancer agents. These biologics have a dual effect: they act on tumor cells and impede their proliferation. On the contrary, because P2X7R, which originated in immune cells, is involved in a variety of immune responses, the use of biologics targeting P2X7R may result in unpredictable and adverse side effects that impair the anti-tumor immune response while promoting tumor growth and metastasis (Bai et al., 2023). As a result, it is critical to understand the mechanism and duration of P2X7R in CRC, conduct additional research on the theoretical basis of P2X7R-targeted therapy for CRC, and develop safe and effective P2X7R-targeted therapy options.

Although the dual role of HMGB1 in tumors remains a mystery, it may represent a promising therapeutic strategy. Under stress conditions, musashi-2 (MSI2) enhances the production, nuclear-to-protoplasmic transport, and extracellular release of DAMP-HMGB1 in CRC cells. A study demonstrated that MSI2 may improve the prognosis of CRC patients by modifying the tumour immunological milieu through post-translational modifications of HMGB1, potentially offering a unique treatment approach for CRC immunotherapy (Meng et al., 2024). However, the complex and dual-faced role of HMGB1 in cancer requires caution in pursuing it as a target for immunotherapy.

### 5.4 Challenges and future directions

With the development of EV purification and omics analysis methods, a large number of IBD exosome biomarkers, including proteins and nucleotides, have been reported. Although miRNA/miRNA features are currently considered a prospective biomarker for CRC and other gastrointestinal cancers and inflammatory gastrointestinal diseases, some reports suggest that most detected miRNAs are not disease-specific (Pritchard et al., 2012). Although publications demonstrating EV miRNA characteristics in cancer have grown exponentially over the past decade, the clinical applicability remains limited due to the lack of endogenous controls to normalize circulating EV miRNA levels and conflicting study results. Currently, no technology can conveniently and reliably purify EVs from clinical samples with high efficiency and yield, which unfortunately restricts clinical development. There is also a lack of studies on screening EV markers specific to different cells or tissues, and the sorting mechanisms of EVs in cells remain unclear. Therefore, several issues regarding the isolation, preservation, and application of EVs require further research.

Regarding modified EVs as drug-delivery vehicles, exosome-mediated therapy has been the focus of study across numerous disciplines, and multiple preliminary clinical trials have been conducted (Rädler et al., 2023). A fundamental gap exists in the understanding of their *in vivo* distribution, mechanisms of action, and toxicological profile. To start with, the time to produce exosomes, the difficulty and cost of large-scale cell cultivation, and the cell’s secretory ability all influence exosome production. Currently, industrial production remains at a low output level, representing a significant obstacle that must be addressed. Moreover, the efficacy of exosomes for cargo delivery is limited by their pre-existing, complex composition, which restricts the amount of foreign material they can carry. Therefore, it is necessary to develop exosome engineering techniques to improve the payload capacity. The lack of sensitive and effective methods for exosome analysis makes the separation of homogeneous exosomes from heterogeneous exosomal populations technically difficult, thereby hampering the development of exosome-based treatment strategies. Due to the complexity of the pathological microenvironment, the efficacy of EV may be influenced by factors such as the degree of inflammation and dysbiosis. Although current clinical studies demonstrate that EV therapy generally exhibits good safety with a low incidence of serious adverse events, its long-term safety still requires further data validation. Furthermore, the traits of EV drugs are significantly influenced by production materials and process parameters. However, there is currently no unified global technical evaluation guideline for EV drugs, which poses challenges for regulatory agencies during the review process. However, if innovative approaches are developed to overcome these limitations, a new era of IBD and associated CRC therapies will emerge. The high biocompatibility of exosomes provides clinicians with more opportunities to create safe targeted therapies for the treatment and management of IBD and CRC.

## 6 Conclusion

In recent years, the importance of EVs in IBD and CRC has grown significantly. The involvement of EV in promoting intestinal inflammation and cancer progression through intercellular communication has been widely recognized. These EVs not only reshape the gut microenvironment, driving the persistence and amplification of chronic inflammation, but also act as a bridge in the progression and metastasis of cancer. Looking ahead, intervention strategies targeting EVs and the novel inflammatory mediators they load show great promise. In diagnostics, detecting EVs and their specific cargos in bodily fluids holds promise as non-invasive, highly sensitive biomarkers. In therapeutics, an oral targeted delivery system based on plant EVs has been established to address the low oral bioavailability of protein or peptide drugs. Engineered EVs can serve as precision drug delivery systems to target and block critical pro-inflammatory or oncogenic pathways. The development of drugs that inhibit the secretion or function of pathogenic EVs could lead to new therapeutic techniques. These techniques allow for safe, effective, and precise targeting in the treatment and management of IBD and CRC. However, challenges remain and will hopefully be overcome through focused efforts. For instance, the strategy for scalable production of high‐quality EVs. Stimulating MSC cultures with inflammatory cytokines and culturing in 3D aggregates under hypoxic conditions significantly enhances EV secretion and confers improved immunomodulatory potential. The transition from preclinical to clinical trials must be facilitated, and standardized specifications for the production and quality control of electric vehicles must be established to ensure consistency and safety. The use of EVs as therapeutic targets or as drug carriers targeting novel inflammatory mediators may soon open new avenues for the clinical management of malignant diseases.
